# A cross-sectional study on feather cover damage in Canadian laying hens in non-cage housing systems

**DOI:** 10.1186/s12917-019-2168-2

**Published:** 2019-12-03

**Authors:** Caitlin Decina, Olaf Berke, Nienke van Staaveren, Christine F. Baes, Tina M. Widowski, Alexandra Harlander-Matauschek

**Affiliations:** 1 Department of Population Medicine, Ontario Veterinary College, University of Guelph, 50 Stone Road E, Guelph, Ontario N1G 2W1 Canada; 2 Department of Animal Biosciences, Ontario Agricultural College, University of Guelph, 50 Stone Road E, Guelph, Ontario N1G 2W1 Canada

**Keywords:** Chicken, Feather pecking, Aviary, Floor system, Welfare

## Abstract

**Background:**

Feather damage (FD) resulting from feather pecking remains a concern in non-cage housing systems for laying hens worldwide. This study aimed to identify bird-, housing-, and management-related factors associated with FD in non-cage housing systems as the egg production sector phases out the conventional cage system in Canada. A survey on housing and management practices was developed and distributed to 122 laying hen farms where 39 respondents provided information on non-cage flocks. Farmers visually assessed 50 birds throughout the barn for FD using a 0–2 scoring scale according to severity. Prevalence of FD was calculated as the percentage of birds with any form of FD (score > 0). Multivariable linear regression modeling was used to identify factors associated with FD prevalence.

**Results:**

Six variables were included in the final model and accounted for 64% of the variation in FD between farms. FD prevalence was higher with increasing flock age (0.9% ± 0.29) and when birds were housed in all wire/slatted barns compared with all litter barns (37.6% ± 13.1). Additionally, FD prevalence tended to be higher in barns with manure removal only after depopulation (20.1% ± 10.70). Enrichment also tended to be associated with higher FD (19.1% ± 8.04), possibly indicating that it was provided after FD was observed as a control measure, or, was not efficient in reducing the development of FD.

**Conclusions:**

These findings emphasize the role of litter provision and management (e.g., manure removal effects on air quality), and its potential impact on FD among laying hens in non-cage housing systems in Canada. Further longitudinal and/or intervention studies are needed to assess the potential of the identified factors to function as a management strategy to prevent or reduce FD in non-cage housed laying hens.

## Background

Canadian egg production has grown over the past 11 years, with a 4.1% increase from 2016 to 2017 in the sale of table eggs – a trend predicted to continue throughout 2018 due to consumers’ positive outlook on eggs and their associated health benefits [1]. With this growth in egg consumption comes a growing consumer interest in enhanced animal welfare standards and a market for cage-free specialty eggs (e.g., free-run, free-range, organic, and nutrient-enhanced). This interest is demonstrated, for example, by a study in British Columbia in which consumption of specialty eggs rose from a combined 8 to 33% for free-range eggs and 12% for organic eggs over a 2-year period [2]. Canada’s major grocers and other food corporations such as McDonald’s® and Tim Hortons® have also contributed to this shift, pledging to buy only cage-free eggs by 2025 [3].

In light of changing market trends, Canadian egg farming is transitioning away from conventional cage housing of hens and into furnished cage and non-cage systems, such as single-tier floor systems and multi-tier aviaries [4]. Though these housing systems offer birds the space and resources to better perform natural behaviours, they pose welfare risks in other ways, most notably a greater opportunity for feather pecking (FP) behaviour and resulting feather damage (FD). FP is the action by which hens peck or pluck at, and sometimes eat the feathers of their conspecifics, causing FD (feathers that are broken, deformed, or deviate from a smooth and intact state) and feather loss typically on the back/rump, vent, and tail areas [5–7]. Loss of feather cover can eventually progress to tissue pecking and mortality due to cannibalism [5].

Feathers are integument features unique to birds and are critical for survival in wild birds. They are smooth, flexible structures largely composed of beta-keratin protein [8]. The various feather types distributed over the body enable birds to carry out locomotive behaviours such as flight or wing-flapping to escape predators and navigate the environment [9, 10], reproductive and social behaviours such as mating displays and shows of aggression through feather raising [8], as well as thermoregulatory behaviours as feathers enable birds to stay warm and dry by providing insulation and a waterproof exterior [11]. In the wild, feathers are kept in good shape with a large portion of the day being dedicated to maintaining their integrity through preening i.e., cleaning, restoring their structure, and applying preen oil [8]. Feathers, much like hair and nails, when damaged cannot repair themselves; thus old feathers get shed through normal preening and replaced through the renewal process of molting [8]. Replacement of feathers can be an immense metabolic stressor, including the replacement of up to 30% of a bird’s body mass [reviewed by [12]] and a basal metabolic rate which can double during molting in avian species [13].

Healthy and functional feather cover is just as important for domestic birds as for wild birds but is much harder to maintain for hens kept in modern housing systems when FP is present. Feather cover damage due to the pecking and pulling out of feathers by other birds represents one of the more common and frustrating challenges in birds kept in intensive production systems [14]. Epidemiological studies in Europe have shown that up to 80% of non-cage flocks exhibit severe FP and FD [15–17]. The most well-accepted hypothesis as to the origin of FP is that it is a redirected foraging/feeding behaviour [18] induced by the stress and frustration of living in a barren environment, but it is ultimately multifactorial, including environmental, nutritional, psychological, and genetic factors [19, 20].

Feather loss and FD undoubtedly cause bird discomfort and present a significant welfare issue. In addition to health and well-being implications of poor feather cover, images of birds without feathers do not meet consumer expectations and increase the risk that consumers will lose trust in egg farming and the livestock food system as a whole [21]. Consumers are increasingly interested in food production practices, with a strong relationship between transparency and trust [22]. A 2017 survey indicated that farmers are those held most responsible for demonstrating trust-building transparency in regards to animal well-being [22]. Furthermore, not having a fully feathered flock can affect farmers through poor financial return due to flock health issues or negative consumer perceptions, but also in terms of diminished job satisfaction due to the daily visual impact of compromised bird welfare [23].

Elucidating the contributing factors leading to FD is thus essential for providing good welfare to millions of hens, but also for fostering continued trust between egg farmers and consumers, and consequently, sustainability within the egg farming community. Though numerous epidemiological studies have been conducted in Europe, to the best of the researchers’ knowledge, no large-scale investigations into FD have been done in alternative housing systems in North America. This study was part of a project to investigate FD in the Canadian context where climate, feeding, and management practices can differ considerably from those in Europe and across regions within Canada. Previously, we presented the methodology used to assess FD by farmers [24] as well as research findings of the project in the context of furnished cage systems [25]. In this paper, we present associations between FD and different aspects of management, environment, health, and genetics, which were identified in an effort to provide farmers with strategies to prevent or control FD.

## Results

### Response rate

Survey packages were distributed to 122 farms with laying hens in alternative housing systems. A total of 64 returned packages were received (52.5% response rate) detailing information for 65 flocks.

### General flock information

Thirty-nine of the 65 flocks were housed in non-cage systems (65%), of which 17 flocks were housed in single-tier floor systems (43.6%), and 22 flocks were housed in multi-tier aviary systems (56.4%). A detailed description of study flocks and their management, including their geographic distribution, is provided in van Staaveren et al. [26]. Descriptive statistics for flock size, flock age, and FD prevalence of the non-cage flocks are presented in Table 1. All flocks were beak-treated at day 1 in the hatchery using an infrared laser. With respect to feather colour, 32% of flocks were white-feathered and 68% were brown-feathered. Within the 30 out of 39 flocks that had available breed information, 60.0% were a Lohmann strain, while other strains included Dekalb (6.7%), Hy-line (16.7%), ISA (10.0%), and Novogen (6.7%).

**Table 1:** Descriptive characteristics of 39 laying hen flocks housed in non-cage systems

	N	Mean (SD)	Median (Range)
Flock age (wks)	39	46.1 (13.87)	45.0 (19–68)
Flock size	37	13,945 (10,949.73)	11,950.0 (119–41,478)
FD prevalence (%)	1950^a^	25.9 (31.70)	10.0 (0–100)

^a^Total number of birds scored for FD (39 × 50)

### Univariable analysis of factors in non-cage systems

Variables associated at a liberal significance level (α = 0.25), or biologically relevant with FD in non-cage laying flocks at the univariable level of analysis are presented in Table 2. These variables were factors related to farmer characteristics, flock characteristics, housing features, litter management, rearing management, nutrition, and feeding and lighting practices (Table 2).

**Table 2:** Explanatory variables (*P* ≤ 0.25) associated with feather damage (FD) at the univariable analysis level

Explanatory Variable	N^a^ (%)	Coefficient	*P*-value
Farmer experience			
≤ 10 years	19 (48.7)	Referent	
More than 10 years	20 (51.3)	−14.57	0.1540
Flock age (weeks)	39 (100)	0.91	0.0118
Feather colour			
White	12 (31.6)	Referent	
Brown	26 (68.4)	14.56	0.1834
Housing system			
Single-tier/floor	17 (43.6)	Referent	
Multi-tier	22 (56.4)	−22.50	0.0259
No. of system levels/tiers			
1 tier	16 (42.1)	Referent	
2 tiers	8 (21.1)	−29.50	0.0306
≥ 3 tiers	14 (36.8)	−21.50	0.0601
Enrichment			
Yes	14 (36.8)	Referent	
No	24 (63.2)	−25.43	0.0159
Floor type			
All litter	8 (21.6)	Referent	
Combination All wire/slatted	21(56.8) 8 (21.6)	8.62 42.25	0.4797 0.0063
Proportion of litter			
No litter	8 (22.2)	Referent	
≤ 1/3 litter	9 (25.0)	−37.03	0.0117
> 1/3 litter	19 (52.8)	− 37.47	0.0041
Litter type			
No litter	8 (22.2)	Referent	
Sawdust or sand	7 (19.4)	−36.39	0.0260
Wood shavings or straw	14 (38.9)	−35.11	0.0130
Manure	7 (19.4)	− 36.39	0.0260
Litter replacement			
Yes	9 (24.3)	Referent	
No	20 (54.1)	−17.53	0.1316
No litter	8 (21.6)	23.92	0.0907
Raking of litter			
No	18 (51.4)	Referent	
Yes	9 (25.7)	−5.78	0.6370
No litter	8 (22.9)	34.14	0.0109
Farmer visit during rear			
Yes	24 (64.9)	Referent	
No	13 (35.1)	13.91	0.2129
Housing type in rear			
Single-tier	20 (54.0)	Referent	
Multi-tier	17 (46.0)	−22.05	0.0326
Matched perches in rear & lay			
Yes	23 (60.5)	−17.33	0.1016
No	15 (39.5)	Referent	
Matched litter in rear & lay			
Yes	20 (52.6)	Referent	
No	18 (47.4)	22.97	0.0242
Manure belt frequency			
> 3x per week	8 (21.6)	Referent	
1-2x per week	17 (46.0)	12.35	0.3376
End of flock	12 (32.4)	35.83	0.0121
Flock health plan in place			
Yes	11 (33.3)	Referent	
No	22 (66.7)	13.73	0.2174
No. of diet changes			
≤ 1 change	13 (34.2)	Referent	
2–3 changes	14 (36.8)	18.31	0.1390
≥4 changes	11 (29.0)	20.85	0.1140
Insoluble grit in diet			
Yes	7 (18.9)	Referent	
No	30 (81.1)	14.29	0.2933
Insoluble fibre in diet			
Yes	13 (35.1)	Referent	
No	24 (64.9)	−15.31	0.1672
Animal by-product in diet			
Yes	6 (17.6)	Referent	
No	28 (82.4)	29.33	0.0463
Dawn/dusk phases			
Yes	30 (76.9)	Referent	
No	9 (23.1)	18.62	0.1236
Dawn/dusk phase method			
All automatic dimmed	15 (38.5)	Referent	
Gradual dim by area	15 (38.5)	−12.80	0.2653
No dawn/dusk	9 (23.1)	12.22	0.3557
Light intensity			
≤ 10 lx	13 (44.8)	Referent	
> 10 lx	16 (55.2)	−26.78	0.0162

^a^Number of flocks in which a response was provided

### Linear regression analysis for non-cage systems

Floor type, the frequency of manure removal/manure belt operation, flock age, enrichment provision, matching of rearing and laying environment by providing litter substrate, and provision of a dawn/dusk period were included in the final model and accounted for 64% of the variation in FD between flocks (Table 3). “Age”, “floor type” (with the largest effect contributed by all wire/slatted barns), and “provision of enrichment” were significantly associated with higher FD. Additionally, FD prevalence tended to be higher with decreasing “manure belt frequency” (with the largest effect when manure was removed only at the end of lay). Not matching litter in both rear and lay, as well as not providing a dawn/dusk phase were positively associated with FD however, the associations were not significant (Table 3).

**Table 3:** Final linear regression model for feather damage prevalence in non-cage laying hen flocks

Variable	Coefficient	SE	*P*-value
Intercept	−18.28	12.528	
Flock age (centered)	0.91	0.293	0.0017
Floor type			< 0.001
All litter	Referent		
Combination	6.50	10.789	
All wire/slatted	37.61	13.065	
Manure belt frequency			0.0151
>3x per week	Referent		
1-2x per week	12.95	9.718	
End of flock only	20.13	10.702	
Enrichment			0.0586
Yes	19.06	8.036	
No	Referent		
Matching of litter^a^			0.2058
Yes	Referent		
No	14.09	9.543	
Dawn/Dusk period			0.1086
Yes	Referent		
No	15.00	9.000	

*α* = 0.05, adjusted *R*^*2*^ = 0.6407, *P* < 0.001, *N* = 39

^a^Matching of litter conditions in both rearing and laying periods of flock’s life

## Discussion

Using a nationally distributed questionnaire and the collection of FD scores from sampled flocks, management, environmental, and genetic factors associated with FD outcomes in laying hen flocks housed in non-conventional cage housing systems were assessed. On average, approximately one quarter of the birds within these flocks exhibited some form of FD, either moderate or severe, and factors most strongly associated with FD included increasing flock age, housing with all wire or slatted floors, manure removal only at the end of production, and provision of enrichment material.

The prevalence of FD found among participating flocks was approximately 26% (95% CI: 15.6–36.2%). This is likely an underestimation of the true prevalence of FD typically exhibited on farms in Canada since the surveyed flocks included some that were relatively young or newly brought into lay. Research on FP behaviour and resultant FD has consistently shown that both increase as birds age [27–29]. This too is illustrated in the findings of the current study in that a small but positive association between the age of a flock and the level of FD was observed. The flocks surveyed in this study were not uniform in age when scored. While younger flocks may be at risk in terms of FP, they may not have begun to show signs of FD at the time of scoring. Had the study surveyed all flocks at the same age in the middle of the production period, median and mean FD prevalence would have likely been greater in value. Previous epidemiological studies in which laying flocks were scored at the same week of age or within a specific age range past 30 weeks, such as that performed in Sweden by Gunnarsson et al. [30], reported damage on the back area in a median of 62% of birds. In the Netherlands, Bestman and Wagenaar [31] found moderate or severe FD in 71% of birds, and more recently, de Haas et al. [17] found 49% of flocks displayed FD.

The hen integument is damaged in all types of housing systems [32, 33]. Research suggests that FP behaviour is initiated by a small percentage of birds and proceeds to spread throughout a flock [34, 35]. Therefore, housing of large flocks in non-cage systems can contribute to an increased prevalence of FD [36, 37]. In comparison, Decina et al. [25] found a FD prevalence of 21.9% in furnished cages compared to the 25.9% prevalence reported here in non-cage systems, which was not different. When comparing the proportion of birds with FD between flocks in conventional cages, furnished cages, free-run barns, and free-range systems, Sherwin et al. [32] found proportions of birds with FD within each system (24.7, 24.9, 26.9, and 15.5%, respectively). However, while the free-range system had the lowest prevalence, they similarly reported no differences in FD prevalence between the furnished cage and barn system.

Floor type had the largest effect on FD, largely due to the effect of all wire or slatted floors, correlated with an increase in FD of approximately 38% in such flocks. Other epidemiological studies have similarly found associations with absence of litter and increased FP activity or FD, such as an absence of loose litter at the end of lay [38] and restriction to the slatted area during nest box training (severe FP was 24 times more likely) [15]. This profound effect is also in line with the generally accepted notion that FP arises from a lack of foraging substrate [18, 39, 40] and the research demonstrating how chicks and pullets reared without suitable litter material show severe FP and poor plumage later in life [41–43].

Low frequency of manure belt running for manure removal was the third factor associated with greater FD. Removal of manure only at the end of the production cycle contributed the most to this effect in that flocks were estimated to exhibit 20% more FD compared to those where manure was cleared more than three times per week. This factor is likely an indicator of air quality, where less frequent manure removal can contribute to increased levels of ammonia in the barn. Concentrations of ammonia tend to be higher in housing systems with manure composting inside the facility compared to systems with regular manure removal, such as manure belts [37]. This has been found in numerous studies when measuring ammonia concentrations in cage systems compared to free-run floor and aviary systems (as reviewed by [44]). Birds find ammonia aversive above concentrations of 20-25 ppm, where it can impair health and reduce immune function [44]. Ammonia at this level is also aversive to barn staff and can compromise their health [45], leading to reduced care and detection of welfare issues within the flock when workers are reluctant to enter the barn. Poor air quality as a general irritant and source of stress plays into the multifactorial nature of FP behaviour, as found by Drake et al. [46] where FD increased with higher carbon dioxide and ammonia in early lay. It is important to note that data was collected in the autumn and early winter months of October to December when ventilation begins to be reduced to conserve warmth in the barn. Decreased ventilation in colder weather paired with humid conditions inside the barn can increase litter moisture and therefore provide a better environment for bacteria to produce ammonia gas [47], a factor which may have influence here.

Removing manure from the barn only at the end of a flock’s cycle can additionally impact air quality in litter systems due to high dust levels when the litter is not changed, and there is poor ventilation of the facility. Dust is contributed by bedding material, feed, dry manure, skin cells, and feathers [48]. Dust and gasses can harm the respiratory system, such as through the loss of cilia needed to clear debris from the upper respiratory tract [49], as well as macro- and microscopic lesions throughout the trachea, lungs, and air sacs [50]. Birds are then predisposed to secondary respiratory diseases caused by bacterial, viral, and fungal infections when the mucosa is compromised [50, 51]. When poor air quality from a multitude of sources leads to negative health outcomes, it contributes to the stress birds experience in these systems and thus increases the risk of FP behaviour and resultant FD.

Lastly, the provision of enrichment in non-cage systems showed a tendency of association with increased FD and was estimated to promote 19% more FD compared to flocks without enrichment. This finding is in opposition to the existing literature – additional foraging opportunities afforded by enrichments such as pecking blocks, hay bales, and hanging objects are viewed as effective methods of FP and FD prevention, especially during rearing [52–54]. It is possible here that if birds were reared in a non-enriched environment, provision of these enrichments during the laying period were ineffective at preventing or minimizing FD, as suggested by Glatz [55]; however the questionnaire did not capture information on enrichment during rearing so this issue cannot be further investigated here. Additionally, producers that have regular issues with FP may have been the ones to provide enrichment. Within the questionnaire, follow-up questions regarding enrichment use revealed that some farms only provided enrichment in response to FD already observed in the flock. Therefore, the effect of enrichment for non-cage flocks should be interpreted with some caution.

It should be noted that this was an exploratory study, as it is the first of its kind in Canada, and thus the *p*-values exhibited should be considered exploratory [56]. Further investigation is needed regarding the impact of factors discussed here. Additionally, no age restrictions were imposed on flocks in lay in this study. It should therefore be recognized that the factors investigated may not have yet reflected their impact on feather cover when the feather assessment was performed for certain flocks newly brought into lay. It should also be acknowledged that data from all provinces were analyzed together; thus, any regional differences in farm practices, which were not distinguished by stratification, could have influence on the results found here. Furthermore, much of the literature comprises studies conducted in Europe where some flocks were not beak-trimmed, and where free-range and organic farming is more common practice than in Canada. Different methods of FD scoring regarding scales used and number of body regions scored also make comparison among study findings difficult.

## Conclusion

Overall, a FD prevalence of approximately 26% was found in a survey of laying hen flocks across Canada, indicating that FP activity is a problem in non-cage systems within the country. Reduced feather cover has important implications for bird welfare (thermoregulation, housing navigation difficulties, and susceptibility to injury), as well as egg farmers (economic losses, low morale or motivation, reduced public support), and consumers (loss of trust in farming practices and animal caretaking). The investigation of housing, management, and genetic factors related to FD indicated that providing birds the opportunity to forage and dustbathe in litter areas in barns continues to be an important element in reducing FD in laying flocks. Cleanliness of the floor area, amount of manure in the barn, and air quality related to these factors should be considered in barns where manure is not removed until the end of lay; such farms could see FD improvement with more frequent manure removal and/or replacement of the litter. Finally, despite contrasting findings of the effect of enrichment on FD outcomes, diversity of the environment through pecking objects and toys is still encouraged for use in the laying barn while attention should be given to enrichment during rearing as well. The factors discussed here would benefit from a longitudinal follow-up study to further investigate the impact of management changes in FD prevention and to better inform the egg farming community on how to prevent FD as more flocks enter alternative housing systems.

## Methods

Study objectives were achieved in three steps: 1) the design of a simplified and easy to use yet comprehensive FD scoring system that does not require handling to assess FD prevalence on-farm, 2) the design of a questionnaire about housing and management practices distributed to egg farmers currently using alternative systems across the country, and 3) linking farm characteristics and practices with the occurrence of FD through regression modelling.

### Feather damage (FD) scoring system

Farmers scored FD in their flock on a severity scale (Table 4) using a devised visual scoring system [24] adapted from previously validated scoring schemes [57–60]. Here, FD encompasses both the destruction of feathers and their loss, while the range of scores covered both good and poor feather condition along with an intermediate score for birds not severely affected by FD. The back/rump of the birds was inspected as this is a frequently targeted region of the body where damage typically reflects FP [28, 61]. The newly developed, simplified scoring system prioritized ease of use and time efficiency to encourage farmer participation.

**Table 4:** Scoring system used by farmers to evaluate feather damage present in their flock

Score	Body condition
0	Intact feather cover, no or slight wear, only single feathers missing
1	Damaged feathers (worn/deformed) or bald patch visible ≤ a $2 coin
2	At least one bald patch visible that is > a $2 coin

Scoring was based on the back/rump area. A Canadian $2 coin is 28 mm in diameter

For the recording of FD scores, farmers were instructed to score a sample of 50 birds selected proportionately from all sections of the barn, as described in previous studies [17, 59, 62]. Detailed and illustrated instructions were provided to assist farmers with selection of birds from different tiers/rows, slatted areas, and litter areas, depending on the housing system. Additionally, instructions included full-colour photographs of white- and brown-feathered birds representing the different scoring categories.

### Questionnaire on housing and management practices

The questionnaire for laying hen farmers was based on a study by Lambton et al. [29], where associations between FP in laying hens and management and environmental factors in alternative systems was investigated. The current questionnaire was tailored using the research team’s expertise to be specific to current practices and standards in Canada [4]. Feedback was sought from federal and provincial egg boards, as well as commercial farmers, to receive input on how well questions reflected commercial settings, to determine if there were discrepancies in how questions would be interpreted, and to gauge overall comprehensiveness, i.e. whether the subject areas being asked about were sufficient, needed further inquiry, or could be pared down for better conciseness. The questionnaire covered the broad areas of flock and bird characteristics, housing features, litter management, flock health, staff duties, rearing history, diet, lighting, and air quality (Table 5). The questionnaire consisted of a mix of open-ended and closed questions with multiple answer options (see Additional file 1), and both English and French versions were made available.

**Table 5:** Housing and management information about a farmer’s current laying hen flock collected through self-administered questionnaire

General Information	Date Years of farming experience Province Farm size
Flock Information	Hatchery & rearing farm birds came from Date of placement Age of placement Current flock age Flock size at placement & current size
Housing Features	Housing system used No. of system tiers Manufacturer & model Age of system Stocking density Perches (availability, height, space) Nests (availability, type, location) Drinker & feeder type Enrichment (types, age of access, motivation for use)
Litter Management	Floor type/proportion of litter Type of material, depth, maintenance Age of access Restriction practices Supplemental foraging material
Bird Characteristics	Feather colour Breed
Rearing and Placement	Visitation of pullet flock Home-rearing vs. supplier, integration of flocks yes/no Pullet housing system Beak trimming (yes/no, age, method, length) Condition on arrival Matching of environmental conditions
Flock Health	Inspection (frequency, duration, no. of workers, route, observations) Feather pecking (if it had been observed, body area, at what age, any management changes in response) Flock behaviour in response to workers Biosecurity measures Vaccination & instances of illness Mortality (percentage & main causes)
Diet	Feed structure, supplier, availability, supplements Feeding frequency & special practices (midnight feeding) Diet changes System breakdowns
Lighting	Type, hours of light, intensity Dawn/dusk period (yes/no) & method
Air quality	Type of ventilation Temperature, humidity, ammonia concentration, dust levels Manure removal frequency
Outdoor Access	Type of access (veranda vs. range area), age of access Range (size, use, quality) Popholes (number, distribution throughout barn) Outdoor area rotation
Productivity	Age at start of lay No. of eggs collected per day, percentage of floor eggs Performance compared to breed standards Current & peak production figures

### Questionnaire distribution

Questionnaire distribution followed the procedures described by Decina et al. [25]. In brief, packages containing all documents (i.e., layer questionnaire, feather cover damage scoring guide, scoring sheets, cover letter, and return-addressed envelope) were posted to participating producers and also made available via Qualtrics® online survey software [63]. Both types of administration were used in order to accommodate groups that favour the ease and speed of an online survey format, as well as groups that may not have reliable access to the Internet or refrain from its use. Participants provided their consent through the return of the questionnaire in agreement with the Tri-Council Policy Statement: Ethical Conduct for Research Involving Humans – TCPS 2 (2018). This study was approved by the University of Guelph Research Ethics Board (REB17–06-010).

Distribution was facilitated through the provincial egg boards to target egg producers that housed flocks in non-cage housing systems and to ensure participant privacy while achieving geographic proportionality. Each package was assigned a 3-digit numeric code in order to ensure all package documents from a participant would remain together throughout the analysis. The data collection period ran from October to December 2017 with reminders sent out by the egg boards 2–4 weeks after initial distribution and two weeks before the end of data collection.

### Statistical methods

Statistical analysis was conducted similarly as for the furnished cage flocks as described by Decina et al. [25], but with a focus on the non-cage flocks. In brief, the percentage of birds with a FD score greater than zero was calculated to estimate the FD prevalence within a flock. Associations between FD prevalence and the different factors investigated through the questionnaire were analyzed using R version 3.4.3 “Kite-Eating Tree” [64] in combination with RStudio [65].

#### Model building

Double manual entry of questionnaire responses was used to limit errors. Variables were screened for excessive missing values (> 50% of responses missing) or insufficient variation (e.g. a proportion of responses within one category of approximately > 0.85), and those were subsequently excluded from further investigation. Several variables were retrospectively collapsed to avoid rare or unobserved categories. For example, due to only a small proportion of farmers indicating different, specific breeds, this variable could not be included, and instead, feather colour was used as the closest proxy. Following this, a total of 61 variables were included in univariable analyses. Variables were examined for collinearity, and associations between each variable were assessed using Spearman rank-correlations (continuous variables) and Pearson’s χ^2^-tests (categorical variables). Twenty-four of the 61 variables reached the criterion of *P* ≤ 0.25, or were considered biologically relevant, and were used as predictor variables for FD prevalence in multivariable analysis using a mixed linear regression model with a forward variable selection approach. The final model included variables that were significant (*P* ≤ 0.05) and/or contributed to a high adjusted R^2^. Relevant interactions between predictor variables were assessed, but none were found to significantly influence the final model. Centering of flock age at 40 weeks was done to allow for a more intuitive interpretation of FD prevalence.

#### Diagnostic procedures

Normality of residuals and homogeneity of variance were graphically inspected using QQ-plots and scatterplots of standardized residuals against fitted values [66]. The Variance Inflation Factor (VIF) was used to assess collinearity, while a boxplot of model residuals was used to check for outliers, and Cook’s distance was used to check for influential data points.

## Supplementary information

**Additional file 1.** Laying farms questionnaire (English versions). Complete questionnaire on housing and management practices distributed to egg farmers with alternative housing systems (English version).

## Data Availability

The dataset used and analyzed during the current study is available from the corresponding author upon reasonable request.

## Abbreviations

FD: Feather Damage
FP: Feather Pecking
