# Supplementary material for: A cross-sectional study on feather cover damage in Canadian laying hens in non-cage housing systems
Source: BMC Vet Res. 2019 Dec 3;15:435. doi: 10.1186/s12917-019-2168-2 (PMC6892204; doi:10.1186/s12917-019-2168-2)
Supplement: Supplementary file 1 — Additional file 1. Laying farms questionnaire (English versions). Complete questionnaire on housing and management practices distributed to egg farmers with alternative housing systems (English version). [file 12917_2019_2168_MOESM1_ESM.pdf]

Towards an understanding of beautiful feather cover in laying hens

A risk factor analysis of feather cover damage in laying hens on Canadian farms

Laying farms
Risk factor questionnaire

Please fill in the following questions to the best of your ability. Please print clearly when writing down your answers or tick the appropriate box as ☒. Only provide one answer (i.e. tick one box) for each question, unless otherwise indicated. There are no right or wrong answers. If you would like to revise your answer, please draw a single horizontal line through the first answer and give your new answer as instructed. If at any point you need more space, you can use a blank sheet and clearly indicate to which question it relates.

1. Background information

Please fill in the current date (dd/mm/yy):____

1. Please indicate how many year(s) of experience you have working in laying hen production:

   ☐ Less than 1 yr ☐ 1-4 yrs ☐ 5-10 yrs ☐ More than 10 yrs
   ☐ I don't know ☐ Prefer not to answer

2. Please indicate in which province/territory your farm is located:

   ☐ Nfld. Lab. ☐ P.E.I. ☐ N.S.
   ☐ N.B. ☐ Quebec ☐ Ontario
   ☐ Manitoba ☐ Sask. ☐ Alberta
   ☐ B.C. ☐ Yukon ☐ N.W.T.
   ☐ Nunavut ☐ I don't know ☐ Prefer not to answer

3. How many barns (a barn is defined as a hen house which houses one flock) are on your farm?

   ☐ 1 ☐ 2 ☐ 3 ☐ 4 ☐ 5 ☐ More than 5
   ☐ I don't know ☐ Prefer not to answer

4. Please give the total number of hens on your farm:

   ☐ Less than 1,000 ☐ 1,000-5,000 ☐ 5,000-10,000
   ☐ 10,000-15,000 ☐ 15,000-20,000 ☐ 20,000-25,000
   ☐ More than 25,000 ☐ I don't know ☐ Prefer not to answer

5. Is your farm certified organic?

   ☐ Yes ☐ No ☐ I don't know ☐ Prefer not to answer

2. Barn information

Please answer the following questions for <u>one barn</u>, which houses the birds in an <u>alternative housing system</u> (i.e. enriched cages, single or multi-tier systems such as free-run or aviary housing, and free-range housing). This is also the barn where you will <u>assess the birds for feather cover damage</u>. Please answer all questions for the <u>current flock</u>.

6. What hatchery did your birds come from?
   ____

7. What rearing farm did your birds come from?
   ____

8. At what date were the birds placed in the barn (dd/mm/yy)?______________________

9. How old were the birds when they were placed in the barn (wks)?__________________

10. What is the current age of the birds in the barn (wks)?__________________________

11. How many birds were placed in the barn in total?__________________________

12. How many birds are currently in the barn?______________________________

13. What are the dimensions of your barn?
    length:_______ft or m *(please circle)**
    width:_______ft or m *(please circle)*
    height:_______ft or m *(please circle)*

**Please circle the unit of measurement you have used to express your answer.*

2.1 Housing

14. Please indicate the housing system of the barn:

☐ Enriched/furnished cage [wire mesh enclosure with perches, nest and scratch area]

☐ Single level indoor system [a non-cage system where birds are housed on only one level/tier e.g. free run]
☐ Single level system – with outdoor access

☐ Multi level indoor system [a non-cage system where birds are housed on multiple levels/tiers e.g. aviary]
☐ Multi level system – with outdoor access

☐ Other. Please specify:__________________________
☐ I don't know ☐ Prefer not to answer

Depending on your answer to question 14, please fill in the appropriate section below.

2.1.1 Enriched/furnished cage housing

(Only fill in this section if you selected the option for 'Enriched/furnished cage' in question 14)

15. How many cage rows are in your barn?

☐ 2 ☐ 3 ☐ 4 ☐ 5 ☐ 6 ☐ More than 6
☐ I don't know ☐ Prefer not to answer

16. How many cage levels/tiers are in your barn?

☐ 2 ☐ 3 ☐ 4 ☐ 5 ☐ 6 ☐ More than 6
☐ I don't know ☐ Prefer not to answer

17. Please indicate the manufacturer of the system:
____

18. Please indicate the model of the system:
____

19. Please indicate the age (years) of the system:

☐ Less than 1 yr old ☐ 1-4 yrs old ☐ 5-10 yrs old
☐ More than 10 yrs old ☐ I don't know ☐ Prefer not to answer

20. How many birds are housed per cage?______________________birds/cage

21. What are the dimensions of the cage?
length:__________in or cm *(please circle)*
width:__________in or cm *(please circle)*
height:__________in or cm *(please circle)*

22. Are perches provided?

☐ Yes ☐ No (Go to question 25) ☐ I don't know ☐ Prefer not to answer

23. Are elevated perches provided at varying heights?

☐ Yes ☐ No ☐ I don't know ☐ Prefer not to answer

24. Is there enough space for all birds to perch at the same time?

☐ Yes ☐ No ☐ I don't know ☐ Prefer not to answer

25. Is a scratch area provided?

☐ Yes ☐ No (Go to question 30) ☐ I don't know ☐ Prefer not to answer

26. What type of material is used for the scratch area?

☐ Smooth plastic ☐ Textured plastic ☐ Artificial grass (e.g. AstroTurf)
☐ Other. Please specify:___________________________________
☐ I don't know ☐ Prefer not to answer

27. Is feed/litter scattered on the scratch area?

☐ Yes ☐ No (Go to question 29) ☐ I don't know ☐ Prefer not to answer

28. How often is feed/litter scattered on the scratch area?

☐ 1x per week ☐ Every other day ☐ 1x per day ☐ 2x per day
☐ Other. Please specify:___________________________________
☐ I don't know ☐ Prefer not to answer

29. How often do you clean the scratch area during a production cycle?

☐ Never ☐ 1x per month ☐ 2x per month ☐ 1x per week
☐ Other. Please specify:___________________________________
☐ I don't know ☐ Prefer not to answer

30. Is a nest area provided?

☐ Yes ☐ No (Go to question 32) ☐ I don't know ☐ Prefer not to answer

31. Does the nest area include any of the following? *Select all that apply.*

☐ Drinkers ☐ Feeders/feeder space ☐ Perches
☐ I don't know ☐ Prefer not to answer

32. Do you provide any form of environmental enrichment to the birds? *Select all that apply.*

☐ None (Go to question 35) ☐ Hanging objects (e.g. bottles, CDs, rope/string)
☐ Alfalfa/mineral blocks ☐ Other. Please specify:______________________
☐ I don't know ☐ Prefer not to answer

33. At what age do you provide access to the environmental enrichment?________________wks

34. Was there a specific event (e.g. cannibalism, high mortality) which motivated you to provide the birds with access to the environmental enrichment?

☐ Yes: __________________________________(please describe)
☐ No (providing enrichment is part of our standard practices)
☐ I don't know ☐ Prefer not to answer

35. Select any of the following if you have made major changes to the infrastructure of the barn (outside of routine changes) since the birds were placed. Please briefly describe the changes. *Select all that apply.*

☐ Cage dimensions:____
☐ Feeders:____
☐ Drinkers:____
☐ Nest area:____
☐ Perches:____
☐ Enrichment:____
☐ Litter/feed (scratchmats):____
☐ Other:____
☐ No changes made ☐ I don't know ☐ Prefer not to answer

Please go to section 2.2 Bird characteristics, p. 10

2.1.2 Non-cage systems
(Only fill in this section if you selected the option for 'Single level' or 'Multilevel system' in question 14)

36. How many levels/tiers are in your barn?

☐ 1 ☐ 2 ☐ 3 ☐ 4 ☐ 5 ☐ 6 ☐ More than 6
☐ I don't know ☐ Prefer not to answer

37. Are resources (e.g. feed, water, nest area, perches) located on each level/tier?

☐ Yes – birds have access to all resources on each level/tier
☐ No – birds do not have access to all resources on each level/tier
☐ I don't know ☐ Prefer not to answer

38. Please indicate the manufacturer of the system:
____

39. Please indicate the model of the system:
____

40. Please indicate the age (years) of the system:

☐ Less than 1 yr old ☐ 1-4 yrs old ☐ 5-10 yrs old
☐ More than 10 yrs old ☐ I don't know ☐ Prefer not to answer

41. Are the birds partitioned or divided into smaller groups using migration fences or wire partitions?

☐ Yes ☐ No (Go to question 44) ☐ I don't know ☐ Prefer not to answer

42. How many sections are the birds divided into?________________________________

43. Are sections of the same size?

☐ Yes ☐ No ☐ I don't know ☐ Prefer not to answer

44. How many birds are housed in each section and what is the space available? *Fill in only section 1 if your barn is not divided into sections.*

Birds in section 1___________ Space of section 1___________ sq.ft or m^2 *(please circle)*
Birds in section 2___________ Space of section 2___________ sq.ft or m^2 *(please circle)*
Birds in section 3___________ Space of section 3___________ sq.ft or m^2 *(please circle)*
Birds in section 4___________ Space of section 4___________ sq.ft or m^2 *(please circle)*

45. Are perches provided?

☐ Yes ☐ No (Go to question 48) ☐ I don't know ☐ Prefer not to answer

46. Are elevated perches provided at varying heights?

☐ Yes ☐ No ☐ I don't know ☐ Prefer not to answer

47. Is there enough space for all birds to perch at the same time?

☐ Yes ☐ No ☐ I don't know ☐ Prefer not to answer

48. Is a nest area provided?

☐ Yes ☐ No (Go to question 52) ☐ I don't know ☐ Prefer not to answer

49. Is the nest area provided as single or group nests?

☐ Single nests ☐ Group nests ☐ I don't know ☐ Prefer not to answer

50. If you have a multi-tier system, are the nests located within the tiers?

☐ Yes ☐ No ☐ I don't know ☐ Prefer not to answer ☐ N/A

51. Is there a perch or platform provided in front of the nest area?

☐ Yes – perch ☐ Yes – platform
☐ No ☐ Other. Please specify:______________________________
☐ I don't know ☐ Prefer not to answer

52. What type of drinkers are used in the barn?

☐ Bell ☐ Nipple
☐ Troughs ☐ Other. Please specify:______________________________
☐ I don't know ☐ Prefer not to answer

53. What type of feeders are used in the barn?

☐ Troughs ☐ Chains
☐ Pan ☐ Hopper
☐ Other. Please specify:____________________________________
☐ I don't know ☐ Prefer not to answer

54. Do you provide any form of environmental enrichment to the birds? *Select all that apply.*

☐ None (Go to question 57) ☐ Hanging objects (e.g. bottles, CDs, rope/string)
☐ Bales of hay/straw – on the floor ☐ Bales of hay/straw – hanging nets
☐ Alfalfa/mineral blocks ☐ Dustbaths (e.g. tire filled with sand or peat)
☐ Other. Please specify:____
☐ I don't know ☐ Prefer not to answer

55. At what age do you provide access to the environmental enrichment?_______________wks

56. Was there a specific event (e.g. cannibalism, high mortality) which motivated you to provide the birds with access to the environmental enrichment?

☐ Yes: ________________________________(please describe)
☐ No (providing enrichment is part of our standard practices)
☐ I don't know ☐ Prefer not to answer

57. Select any of the following if you have made major changes to the infrastructure of the barn (outside of routine changes) since the birds were placed. Please briefly describe the changes. *Select all that apply.*

☐ Sections/partitions:____
☐ Feeders:____
☐ Drinkers:____
☐ Nest area:____
☐ Perches:____
☐ Enrichment:____
☐ Litter:____
☐ Outdoor access/area:____
☐ Other:____
☐ No changes made ☐ I don't know ☐ Prefer not to answer

2.1.2.1 Litter management

58. Please select the description that best applies to your barn:

☐ All litter barn (Go to question 60) ☐ Combination of wire, slats and litter
☐ All wire/slatted barn (Go to Section 2.2 Bird characteristics, p. 10)
☐ I don't know ☐ Prefer not to answer

59. What proportion of the barn is covered with litter?

☐ Less than ⅓ ☐ ⅓ ☐ More than ⅓
☐ I don't know ☐ Prefer not to answer

60. What type of litter is provided? *Select all that apply*.

☐ Sawdust ☐ Sand ☐ Wood shavings
☐ Straw – chopped ☐ Straw – long ☐ Straw - wheat
☐ Straw - barley ☐ Other. Please specify:_________________________________
☐ I don't know ☐ Prefer not to answer

61. At what age did birds first gain access to the litter?_________________________wks of age

62. If birds are restricted from access to litter, during which time of day do you restrict the birds' access? *Select all that apply.*

☐ 06:00-09:00 ☐ 09:00-12:00 ☐ 12:00-15:00
☐ 15:00-18:00 ☐ 18:00-21:00 ☐ Other. Please specify:_______________
☐ N/A ☐ I don't know ☐ Prefer not to answer

63. Do you scatter anything (e.g. grain/grit) to encourage 'working' of the litter?

☐ Yes__________________(please specify what) ☐ No
☐ I don't know ☐ Prefer not to answer

64. What approximate depth is the litter currently kept at?_____________in or cm *(please circle)*

65. Have you replaced/replenished the litter since the birds first gained access to the litter?

☐ Yes, if so how many times?__________ ☐ No
☐ I don't know ☐ Prefer not to answer

66. How often do you rake/fork/rotovate the litter (i.e. breaking up of the litter) for the current flock?

☐ Never ☐ Monthly ☐ Weekly ☐ Daily
☐ Other. Please specify:_______________ ☐ I don't know ☐ Prefer not to answer

2.2 Bird characteristics

67. Please indicate the feather colour of the birds in the barn. *Select all that apply*:

☐ Brown hens ☐ White hens
☐ Silver hens ☐ Other. Please specify:____________________________
☐ I don't know ☐ Prefer not to answer

68. Please indicate the breed(s) of the birds in the barn. *Select all that apply:*

☐ Babcock ☐ Bovans
☐ Dekalb ☐ Hisex
☐ H&N ☐ Hy-Line
☐ ISA ☐ Lohmann
☐ Shaver ☐ Other. Please specify:______________________
☐ I don't know ☐ Prefer not to answer

69. If known, please indicate the specific breed(s) of the birds in the barn. *Select all that apply: Breeds are grouped by feather colour and alphabetic order*

☐ Babcock Brown ☐ Bovans Brown
☐ Dekalb Brown ☐ Hisex Brown
☐ H&N Brown Nick ☐ Hy-Line Brown
☐ ISA Brown ☐ Lohmann Brown Classic
☐ Lohmann Brown Extra ☐ Lohmann Brown Lite
☐ Lohmann Brown Plus ☐ Lohmann Tradition
☐ Shaver Brown

☐ Babcock White ☐ Bovans White
☐ Dekalb White ☐ Hisex White
☐ H&N Crystal Nick ☐ H&N Nick Chick
☐ H&N Super Nick ☐ Hy-Line W-36
☐ Hy-Line W-80 ☐ ISA White
☐ Lohmann LSL Classic ☐ Lohmann LSL Extra
☐ Lohmann LSL Lite ☐ Lohmann LSL Ultra Lite
☐ Shaver White

☐ Dekalb Amberlink ☐ H&N Coral Nick
☐ Hy-Line Silver Brown ☐ Hy-Line Sonia/Gray

☐ Lohmann Dual ☐ Lohmann Sandy
☐ Lohmann Silver ☐ Other. Please specify:______________________
☐ I don't know ☐ Prefer not to answer

2.3 Rearing and placement

70. Where do you source your birds from?

☐ Home-reared ☐ Rearing supplier ☐ I don't know ☐ Prefer not to answer

71. Did you visit the birds during rear?

☐ Yes ☐ No ☐ I don't know ☐ Prefer not to answer

72. Did all birds in the barn come from the same rearing flock?

☐ Yes ☐ No ☐ I don't know ☐ Prefer not to answer

73. In which type of housing were the pullets raised?

☐ Conventional cage [wire mesh enclosure without perches, nest and scratch area]
☐ Enriched/furnished cage [wire mesh enclosure with perches, nest and scratch area]

☐ Single level indoor system [a non-cage system where birds are housed on only one level/tier e.g. free run]
☐ Single level system – with outdoor access

☐ Multi level indoor system [a non-cage system where birds are housed on multiple levels/tiers e.g. aviary]
☐ Multi level system – with outdoor access

☐ Other. Please specify:__________________________________
☐ I don't know ☐ Prefer not to answer

74. Are your birds beak trimmed?

☐ Yes, at__________________days or weeks *(please circle)*
☐ No (Go to question 76) ☐ Retrospectively
☐ Repeat ☐ Other. Please specify:____________________________
☐ I don't know ☐ Prefer not to answer

75. Please indicate what method of beak trimming was used and how much was trimmed:

☐ Hot blade beak trimming: ¼ or ⅓ or ½ of beak length trimmed *(please circle)*
☐ Infrared beak trimming: ¼ or ⅓ or ½ of beak length trimmed *(please circle)*
☐ Other. Please specify:____________________________________
☐ I don't know ☐ Prefer not to answer

76. Did you see any of the following when your birds arrived? *Select all that apply.*

☐ Birds with varying body size
☐ Birds with leg injuries
☐ Birds with blood visible on their body
☐ Birds with feather damage/naked spots on the head
☐ Birds with feather damage/naked spots on the back
☐ Other. Please specify:____________________________
☐ None of the above ☐ I don't know ☐ Prefer not to answer

77. For this flock, did you aim to match the conditions in your barn to the conditions in which birds are kept during rear?

☐ Yes ☐ No (Go to question 79) ☐ I don't know ☐ Prefer not to answer

78. If yes, in what way(s) did you try to match conditions? *Select all that apply.*

☐ Housing type (e.g. cage, free-run, aviary)
☐ Drinker type
☐ Drinker height
☐ Feeder type
☐ Feeder height
☐ Time feeders are run
☐ Feed structure (e.g. mashed, pelleted etc.)
☐ Time lights on and off
☐ Type of light bulbs
☐ Perch access and/or type
☐ Litter access and/or type
☐ Other. Please specify:__________
☐ I don't know
☐ Prefer not to answer

2.4 Flock health

79. How many times per day are the birds inspected at their current age?

☐ 1x ☐ 2x ☐ 3x ☐ 4x ☐ 5x ☐ More than 5x
☐ I don't know ☐ Prefer not to answer

80. Please indicate how many workers inspect the current flock on a daily basis:

☐ 1 ☐ 2 ☐ 3 ☐ 4 ☐ 5 ☐ More than 5
☐ I don't know ☐ Prefer not to answer

81. Do you inspect the top and bottom tier during inspections of the current flock?

☐ Never ☐ Sometimes ☐ About half of the time ☐ Most of the time
☐ Always ☐ I don't know ☐ Prefer not to answer

82. On average how long do you spend inspecting the current flock per day?

☐ Less than 15 min ☐ 15-30 min ☐ 30-45 min ☐ 45-60 min ☐ More than 60 min
☐ I don't know ☐ Prefer not to answer

83. Do you vary the route taken through the house during inspections of the current flock?

☐ Yes ☐ No ☐ I don't know ☐ Prefer not to answer

84. How often do you pick up an individual bird during an inspection of the current flock?

☐ Never ☐ Sometimes ☐ About half of the time ☐ Most of the time
☐ Always ☐ I don't know ☐ Prefer not to answer

85. What do you look for during inspections? *Select all that apply.*

☐ Dead birds ☐ Sick birds
☐ Birds with leg injuries ☐ Birds with blood visible on their body

☐ Birds with feather damage/naked spots on the head
☐ Birds with feather damage/naked spots on the back

☐ Birds with varying body size ☐ Birds are evenly distributed within the cage/barn
☐ Behaviour of the birds ☐ Pile-up/smothering
☐ Vocalization of the birds ☐ Feather eating from the floor

☐ Floor/system eggs (i.e. eggs laid outside of the nest)
☐ Malfunctioning feeders/drinkers
☐ Malfunctioning ventilation/temperature systems
☐ Malfunctioning lighting systems

☐ Litter quality (e.g. wet litter, litter depth)
☐ Feathers in the litter
☐ Other. Please specify:____
☐ I don't know ☐ Prefer not to answer

86. Have you noticed a higher amount of feathers than usual in the litter of this flock?

☐ Yes ☐ No ☐ I don't know ☐ Prefer not to answer

87. Have you noticed birds eating feathers from the floor in this flock?

☐ Yes ☐ No ☐ I don't know ☐ Prefer not to answer

88. Have you seen birds with feather damage/naked spots on the head in the current flock?

☐ Yes, from_________wks of age ☐ No (Go to question 91)
☐ I don't know ☐ Prefer not to answer

89. Please briefly explain why you think this is occurring:
____

90. Have any changes been made in the management of the birds to try and address this? Please briefly describe the changes. *Select all that apply.*

☐ Beak trimming:____
☐ Lower light intensity:____
☐ Changed feed composition:____
☐ Change in stocking density:____
☐ Change in enrichment:____
☐ Change in litter management:____
☐ Other. Please specify:____
☐ No changes made ☐ I don't know ☐ Prefer not to answer

91. Have you seen birds with feather damage/naked spots on the back in the current flock?

☐ Yes, from_________wks of age ☐ No (Go to question 94)
☐ I don't know ☐ Prefer not to answer

92. Please briefly explain why you think this is occurring:
____

93. Have any changes been made in the management of the birds to try and address this? Please briefly describe the changes. *Select all that apply.*

☐ Beak trimming:____________________
☐ Lower light intensity:____________________
☐ Changed feed composition:____________________
☐ Change in stocking density:____________________
☐ Change in enrichment:____________________
☐ Change in litter management:____________________
☐ Other:____________________
☐ No changes made ☐ I don't know ☐ Prefer not to answer

94. How do birds in the current flock react to you when you first enter the barn?

☐ No reaction ☐ Run away ☐ Flighty/pile-up ☐ Crowd around
☐ Other. Please specify:____________________
☐ I don't know ☐ Prefer not to answer

95. How do birds in the current flock react to you when you are standing still?

☐ No reaction ☐ Run away ☐ Flighty/pile-up ☐ Crowd around
☐ Other. Please specify:____________________
☐ I don't know ☐ Prefer not to answer

96. Please indicate how often veterinary practitioners visit the current flock in your laying facility:_______times/year

97. Have you developed and implemented a flock health plan with your vet?

☐ Yes ☐ No ☐ I don't know ☐ Prefer not to answer

98. Did you clean and disinfect the barn before the birds arrived?

☐ Yes ☐ No ☐ I don't know ☐ Prefer not to answer

99. Do you use dedicated clothing and/or clean boot dips for the barn?

☐ Yes ☐ No ☐ I don't know ☐ Prefer not to answer

100. Have you re-vaccinated your birds since placement?

☐ Yes: ____________________________________ (please specify for what)
☐ No
☐ Other. Please specify:___________________________________
☐ I don't know ☐ Prefer not to answer

101. Were any of the following problems observed in the flock since placement? *Select all that apply.*

☐ Avian rhinotracheitis ☐ Coccidiosis
☐ E. coli ☐ Eggbound
☐ Egg peritonitis ☐ Infectious bronchitis
☐ Layer fatigue ☐ Lice
☐ Marek's disease ☐ Mites
☐ Necrotic enteritis ☐ Worms
☐ Other. Please specify:___________________________________
☐ None ☐ I don't know ☐ Prefer not to answer

102. What is the total number of birds that have died since placement?
___________________ birds
___________________% cumulative mortality

103. Please rank the 3 main causes of culling/mortality from the most [1] to the least common [3] *(max 3 options)*:

____Trapped in housing equipment (e.g. caught in feeder)
____Smothering (i.e. pile-up or crowding of birds)
____Heat/cold stress
____Cannibalism (i.e. death due to excessive pecking by other birds)
____Disease/illness. Please specify: ________________________________
____Leg injuries
____Prolapse
____Other. Please specify:___________________________________
☐ I don't know ☐ Prefer not to answer

2.5 Diet and feeding

104. Please indicate the structure of the feed you currently give the birds:

☐ Mashed feed ☐ Pelleted feed
☐ Grains ☐ Crumbs
☐ Other. Please specify:___________________________________
☐ I don't know ☐ Prefer not to answer

105. What is the source of the feed?

☐ Home-milled
☐ Purchased from supplier: ____
(please provide name of diet and company)
☐ Other. Please specify:______________________
☐ I don't know ☐ Prefer not to answer

In order to get more insight into the relationship between nutrition and feather damage, we would like to get a better idea of the composition of your current diet. Please consider sending a <u>picture or copy of the ingredient labels of your diet</u> when returning the questionnaire.

106. Have you changed the diet since the birds were placed in the barn, if so how many times?

☐ No change (Go to question 108) ☐ 1x ☐ 2x ☐ 3x ☐ 4x ☐ More than 4x
☐ I don't know ☐ Prefer not to answer

107. If you changed the diet, did you change the diet gradually?

☐ Yes - gradual change ☐ No - immediate change
☐ Other. Please specify:___________________________________
☐ I don't know ☐ Prefer not to answer

108. How is feed provided in terms of availability?

☐ Ad libitum (available at all times) ☐ Restricted
☐ Other. Please specify:________________ ☐ I don't know
☐ Prefer not to answer

109. Please indicate how often feeders are run:________times/day

110. Do you use midnight feeding for the current flock?

☐ Yes ☐ No ☐ I don’t know ☐ Prefer not to answer

111. Has the feeder system had any breakdowns/problems resulting in feed restriction since the birds were placed? If so, how many times?

☐ Yes, 1-3x ☐ Yes, 4-6x ☐ Yes, more than 6x
☐ No ☐ I don’t know ☐ Prefer not to answer

112. Has the drinker system had any breakdowns/problems resulting in water restriction since the birds were placed? If so, how many times?

☐ Yes, 1-3x ☐ Yes, 4-6x ☐ Yes, more than 6x
☐ No ☐ I don’t know ☐ Prefer not to answer

113. Did/do you provide any of the following feed supplements to the current flock? *Select all that apply.*

☐ Insoluble grit (e.g. sand)
☐ Insoluble fibre (e.g. whole oats, wheat, corn, alfalfa, maize/barley/pea silage etc.)
☐ Oyster shell
☐ Vitamins
☐ Other. Please specify:___________________________________
☐ No supplements provided
☐ I don’t know ☐ Prefer not to answer

114. Do you have any animal by-products in your ration?

☐ Yes ☐ No ☐ I don’t know ☐ Prefer not to answer

2.6 Lighting

115. Please indicate which type of lighting is provided in the barn:

☐ Incandescent ☐ Fluorescent ☐ LED
☐ Other. Please specify:_______________________ ☐ Natural light (i.e. windows)
☐ I don’t know ☐ Prefer not to answer

116. Please indicate the number of hours of light birds are on:________________________h/day
Time on:______________[hh:mm] off:______________[hh:mm]

117. Do you provide a dawn/dusk lighting period for the current flock?

☐ Yes ☐ No (Go to question 119) ☐ I don't know ☐ Prefer not to answer

118. Please indicate how dawn/dusk is achieved:

☐ Natural light (i.e. windows) ☐ All lights dimmed automatically
☐ Gradual dimming by area ☐ Other. Please specify:______________________
☐ I don't know ☐ Prefer not to answer

119. Please indicate what the average light intensity is (lux) at bird height on the lowest level/tier

☐ Less than 5 lux ☐ 5-10 lux ☐ 11-15 lux ☐ 16-20 lux
☐ 21-25 lux ☐ More than 25 lux ☐ I don't know ☐ Prefer not to answer

120. Is light intensity even throughout the barn (i.e. no darker areas)?

☐ Even ☐ Somewhat even ☐ Somewhat uneven
☐ Uneven ☐ I don't know ☐ Prefer not to answer

121. Do you use the management guidelines for your breed of birds for the lighting system?

☐ Yes ☐ No ☐ I don't know ☐ Prefer not to answer

2.7 Air quality

122. Please indicate what type of ventilation system is used

☐ Controlled (fan) ☐ All natural
☐ Mixed (fan and natural) ☐ Other. Please specify:______________________
☐ I don't know ☐ Prefer not to answer

123. Please indicate the temperature (°C) setting in the barn:____________________________

124. Please indicate the relative humidity (%RH) setting in the barn:______________________

125. Has the concentration of ammonia ever reached intolerable levels (above 20-25 ppm, i.e. irritable to eyes or airways) since placement of the birds in the barn?

☐ Yes ☐ No ☐ I don't know ☐ Prefer not to answer

126. How often do you remove the manure from the barn (e.g. by manure belt/scraper(s))?

☐ 1x per day ☐ 3x per week ☐ 2x per week ☐ 1x per week
☐ Other. Please specify:________________________________
☐ I don't know ☐ Prefer not to answer

127. Has the concentration of dust ever reached intolerable levels (i.e. irritable to eyes or airways) since placement of the birds in the barn?

☐ Yes ☐ No ☐ I don't know ☐ Prefer not to answer

128. Do you follow the management guidelines for your breed of birds for air quality?

☐ Yes ☐ No ☐ I don't know ☐ Prefer not to answer

2.8 Outdoor access and range use

(Only fill in this section if you selected an option 'with outdoor access' in question 14)

129. What type of outdoor access do you provide to the birds?

☐ Covered veranda [roof covered barn extension] ☐ Free range area
☐ I don't know ☐ Prefer not to answer

130. Please indicate the age of the birds when they were first allowed outside: ___________wks

131. What is the size of the range birds have access to?__________________________hectare

132. Please indicate the proportion of the range that is regularly used: ___________________%

133. Please indicate the proportion of the birds that use the range: _____________________%

134. Which phrase best describes how your birds use the range?

☐ Stay near the barn
☐ Stay near the outdoor structures (e.g. artificial/natural shelters)
☐ Use less than half of the outdoor area
☐ Use more than half of the outdoor area
☐ Use the entire outdoor area
☐ I don't know ☐ Prefer not to answer

135. What is the number of openings to the range area?

☐ 1-3 ☐ 4-5 ☐ More than 5 ☐ I don't know ☐ Prefer not to answer

136. Are the openings to the range area evenly distributed along the barn?

☐ Yes ☐ No ☐ I don't know ☐ Prefer not to answer

137. Which of the following do you provide to the birds in the outdoor area? *Select all that apply*.

☐ Feed/feeders
☐ Water/drinkers
☐ Artificial shelter (e.g. sheds, old farm equipment)
☐ Natural shelter (e.g. shrubs, trees)
☐ Nest area(s)
☐ Dustbathing area(s)
☐ Perches
☐ None ☐ Other. Please specify:______________________________
☐ I don't know ☐ Prefer not to answer

138. Do you rotate the outdoor area during the time it is in use (during a laying cycle)?

☐ Yes – 1x ☐ Yes – 2x ☐ Yes – 3x ☐ No
☐ Other. Please specify:______________________________
☐ I don't know ☐ Prefer not to answer

139. Do you rotate the outdoor area for each new flock?

☐ Yes ☐ No ☐ I don't know ☐ Prefer not to answer

140. Do you have, or have you had problems with predators in the current flock?

☐ Yes ☐ No ☐ I don't know ☐ Prefer not to answer

2.9 Egg production and flock performance

141. Please indicate the age of the birds when day length was increased: ________________wks

142. When did your birds start lay?

☐ Before 19 wks of age ☐ At 19 wks of age ☐ After 19 wks of age
☐ I don't know ☐ Prefer not to answer

143. How many eggs are collected per day in this barn?_________________________eggs/day

144. What is the percentage of floor and system eggs (i.e. outside the nest) in this barn?______%

145. Do you have a problem with egg eating in this barn?

☐ Yes ☐ No ☐ I don't know ☐ Prefer not to answer

146. Please indicate how performance of the birds compares with the breed standards/targets:

	Below target	On target	Above target	I don't know	Prefer not to answer
No. of eggs produced	☐	☐	☐	☐	☐
Body weight	☐	☐	☐	☐	☐
Feed consumption	☐	☐	☐	☐	☐
Mortality	☐	☐	☐	☐	☐

147. What is the current egg production of the birds?___________________production rate (%)

148. What was the age of the birds at 50% rate of lay?______________________________wks

149. What was the age of the birds at peak production? *Only answer if birds have reached peak production*____wks

150. What was the peak production of the birds? *Only answer if birds have reached peak production*____production rate (%)

151. Please indicate the average feed consumption:__________________________gr/day/bird

3. Demographics and other information

152. Please indicate your gender:

☐ Female ☐ Male ☐ Other. Please specify:____
☐ I don’t know ☐ Prefer not to answer

153. Please indicate your age (years):

☐ 18-24 ☐ 25-34 ☐ 35-44 ☐ 45-54 ☐ 55-64
☐ 65 and above ☐ Prefer not to answer

154. Have you completed specific laying hen-related training/course(s)?

☐ Yes – 1 course/training ☐ Yes – 2 to 3 courses/trainings
☐ Yes – more than 3 courses/trainings ☐ No
☐ I don’t know ☐ Prefer not to answer

155. Please indicate your main sources of information/advice on egg production. *Select all that apply*.

☐ Veterinarians ☐ Laying hen specialists/advisors/inspectors
☐ Other egg producers ☐ Management/co-workers
☐ Industry organizations/meetings/journals
☐ Research organizations/meetings/journals
☐ Internet ☐ Other. Please specify:_________________
☐ I don’t know ☐ Prefer not to answer

156. Would you be willing to participate in future research?

☐ Yes ☐ No ☐ I don’t know
